# Vitamin D Dietary Intake Questionnaire Validation Conducted among Young Polish Women

**DOI:** 10.3390/nu8010036

**Published:** 2016-01-05

**Authors:** Dominika Głąbska, Dominika Guzek, Patrycja Sidor, Dariusz Włodarek

**Affiliations:** 1 Chair of Dietetics, Department of Dietetics, Faculty of Human Nutrition and Consumer Sciences, Warsaw University of Life Sciences WULS—SGGW, Warsaw, Poland—159c Nowoursynowska Str, 02-776 Warsaw, Poland; patrycja_sidor@sggw.pl (P.S.); dariusz_wlodarek@sggw.pl (D.W.); 2 Laboratory of Food Chemistry, Faculty of Human Nutrition and Consumer Sciences, Warsaw University of Life Sciences WULS—SGGW, Warsaw, Poland—159c Nowoursynowska Str, 02-776 Warsaw, Poland; dominika_guzek@sggw.pl

**Keywords:** vitamin D, food frequency questionnaire, validation study, validity, reproducibility, young women

## Abstract

Due to inadequate intake of Vitamin D, identification of individuals characterised by the highest risk of deficiencies is one of the more crucial tasks for public health. The aim of the presented study was to assess the validity and reproducibility of the designed Vitamin D dietary intake questionnaire based on food frequency assessment—VIDEO-FFQ (VItamin D Estimation Only—Food Frequency Questionnaire) in a group of Polish women aged 20–30 years. Seventy-five participants kept a three-day dietary record and filled out the VIDEO-FFQ twice (immediately after the three-day dietary record and after six weeks). The assessment of validity and reproducibility was conducted by verifying standard errors of estimation, median differences, and percentages of individuals classified into tertiles, correlations and Bland-Altman plots. The Vitamin D intake for the majority of the surveyed women was inadequate as over 85% of them were characterised by values of intake lower than 5.0 μg per day. The results allowed concluding that a high accuracy of the VIDEO-FFQ was achieved. The required Bland-Altman index values lower than 5.0% were obtained, confirming satisfactory validity and reproducibility. The VIDEO-FFQ may be deemed a convenient practical tool for the estimation of Vitamin D intake in young women.

## 1. Introduction

For a long time now, the worldwide Vitamin D dietary intake has been observed as inadequate [1]. The report of the European Food Safety Authority [2] indicated that the dietary intake of Vitamin D is commonly too low for women in European countries. The highest Vitamin D dietary intake was observed in Finland (daily average intake of 6.0 μg of cholecalciferol for women) [3] and Sweden (daily average intake of 5.8 μg of cholecalciferol for 55–64-year-old women and 6.1 μg for 65–74-year-old women) [4], which might be attributed to high consumption of oil-rich fish, as well as fortified food products [5]. In other countries, Vitamin D dietary intake is reported to be significantly lower—e.g., in Poland, the daily average cholecalciferol intake was 3.3 μg for women [6]. It was indicated, in the European Nutrition and Health Report [7], that vitamin D intake is generally low in nearly all age groups. Similarly, the *EURopean micronutrient RECommendations Aligned (EURRECA) Network of Excellence Project* revealed Vitamin D to be the nutrient characterised by one of the highest prevalences of inadequate intake in Europe, as inadequacy was stated for over 40% of individuals [8].

In parallel, new evidence has revealed that everyday Vitamin D intake should be even higher than previously recommended, which was reflected in the report of Institute of Medicine [9], indicating 10.0 μg (Estimated Average Requirement—EAR) and 15.0 μg (Recommended Dietary Allowance—RDA) of cholecalciferol as the reference daily intake for adults. However, the interpretation of recommendations specifies that sunlight exposure and the resulting Vitamin D production in skin may affect the necessary dietary intake [10].

Due to the reported inadequate Vitamin D intake, identification of the individuals characterised by the highest risk of Vitamin D deficiencies may be one of the more crucial tasks for public health [11]. As individuals with low intake are simultaneously at an increased risk of Vitamin D deficiency, a suitable quick screening procedure is necessary [12].

The problem of inadequate Vitamin D intake is most serious in the case of young women, as women under 30 years old were characterised by the lowest Vitamin D intake among the all age groups on the basis of the United States NHANES 2003–2006 data [13]. Moreover, for almost 80% of such women, intake values lower than the Adequate Intake (AI) level were observed [13]. Simultaneously, by the age of 30 years, women reach their peak bone mass, maximum bone strength, as well as maximum bone density, and afterwards the initiation of progressive loss of bone mass occurs [14], while Vitamin D significantly influences bone mineral density [15].

The various food frequency questionnaires aimed at assessment of Vitamin D intake were designed and validated in several countries—in the United States of America [16,17,18,19], Canada [20,21], the United Arab Emirates [22], and South Korea [23]. However, it should be noted that specific food products are consumed in each country or geographical region, so such products must always be included in the analysis, and appropriate questionnaires dedicated for the country or region should be designed.

The aim of the presented study was to assess the validity and reproducibility of the designed Vitamin D dietary intake questionnaire based on food frequency assessment—VIDEO-FFQ (VItamin D Estimation Only—Food Frequency Questionnaire) for a group of Polish women aged 20–30 years.

## 2. Experimental Section

The study was conducted according to the guidelines laid down in the Declaration of Helsinki, and all the procedures involving human subjects were approved by the Ethics Committee of the Regional Medical Chamber in Warsaw, Poland (No. 4/08; 7.02.2008).

### 2.1. Vitamin D Dietary Intake Questionnaire (VIDEO-FFQ—VItamin D Estimation Only—Food Frequency Questionnaire)

The VIDEO-FFQ was based on the food frequency assessment, and only food products being sources of Vitamin D were taken into account. The chosen products were those characterised by a content of Vitamin D not lower than 0.01 μg/100 g on the basis of the Polish food composition tables [24]. The selected food products were divided into eight groups encompassing different ranges of Vitamin D content (Table 1). The determined regular serving sizes based on the Polish atlas of portion sizes of food products and dishes [25] were verified during the pilot study. Simultaneously, the most reasonable frequency of consumption was determined and verified for each product during the pilot study. The pilot study was conducted in a group of five young female individuals, who received VIDEO-FFQ, including preliminarily specified portion sizes of food products and dishes, as well as frequencies of consumption. The participants were asked to fill out the questionnaire. Subsequently, products’ and dishes’ portion sizes, as well as frequencies of consumption, were verified and, if needed, changed into more reasonable ones on the basis of the obtained declared numbers of servings.

The average Vitamin D content in a serving was specified for each group of food products, as presented in Table 2. The average Vitamin D content per serving was specified on the basis of the Polish food composition tables developed using reference chemical analysis of food products available on the Polish market [24]. The information regarding Vitamin D quantity in the serving was not included in the VIDEO-FFQ in order not to interfere with the answers.

**Table 1:** The scheme of an applied food frequency questionnaire including food products, serving sizes and frequencies in the VIDEO-FFQ.

Group of Products	Product	Serving Size	Frequency	Number of Servings
Fresh and smoked fish	Salmon, rainbow trout, herring, eel	50 g (deck of cards)	monthly	
	Halibut, mackerel, brook trout, sole, tuna	50 g (deck of cards)	monthly	
	Cod, flounder, plaice, pollock, hake, bass, zander, pike	50 g (deck of cards)	monthly	
Fish products	Herrings, sardines and tuna products	100 g (e.g., 2 rollmopses, small can of tuna, 2/3 of can of herrings)	monthly	
	Other fish products	100 g (e.g., 1/3 of can of fish stew)	monthly	
Dairy products	Milk and milk beverages (yoghurt, kefir, buttermilk, cream)	250 g (1 glass)	weekly	
	Rennet cheese	20 g (1 slice)	weekly	
	Blue and soft penicillium cheese	150 g (1 package)	weekly	
	Feta cheese	15 g (1 slice)	weekly	
	Cottage cheese	50 g (1 thick slice, 2 tablespoons)	weekly	
	Processed cheese	25 g (1 slice, 1 spoon, 1 triangle serving)	weekly	
	Homogenized cheese, dairy dessert	150 g (1 package)	weekly	
	Dairy ice cream	40 g (1 scoop)	monthly	
Eggs	Egg	50 g (1 medium egg)	weekly	
	Egg yolk	20 g (1 yolk)	weekly	
Meat		100 g (palm of small hand)	weekly	
Meat products		15 g (thin slice of ham, 3 slices of sausage)	weekly	
Cereals	White wheat and confectionery bread	35 g (1 slice, small roll)	weekly	
	Cooked egg pasta	100 g of cooked (1 glass)	weekly	
Fats	Butter, butter products, pork fat	5 g (1 teaspoon)	daily	
	Margarine	5 g (1 teaspoon)	daily	

**Table 2:** Vitamin D contents for the single serving sizes of the products specified in the VIDEO-FFQ.

Group of Products	Product	Serving Size	Vitamin D Content per 1 Serving (μg)
Fresh and smoked fish	Salmon	50 g	7.50
	Rainbow trout	50 g	7.80
	Herring	50 g	9.50
	Eel	50 g	15.00
	Halibut	50 g	2.50
	Mackerel	50 g	2.50
	Brook trout	50 g	1.05
	Sole	50 g	4.00
	Tuna	50 g	3.60
	Cod	50 g	0.50
	Flounder	50 g	0.40
	Plaice	50 g	0.40
	Pollock	50 g	0.50
	Hake	50 g	0.50
	Bass	50 g	0.40
	Zander	50 g	0.35
	Pike	50 g	0.45
Fish products	Herrings, sardines and tuna products	100 g	12.36
	Other fish products	100 g	0.93
Dairy products	Milk and milk beverages (yoghurt, kefir, buttermilk, cream)	250 g	0.28
	Rennet cheese	20 g	0.09
	Blue and soft penicillium cheese	150 g	0.29
	Feta cheese	15 g	0.08
	Cottage cheese	50 g	0.08
	Processed cheese	25 g	0.07
	Homogenized cheese, dairy dessert	150 g	0.23
	Dairy ice cream	40 g	0.30
Eggs	Egg	50 g	0.85
	Egg yolk	20 g	0.90
Meat		100 g	0.75
Meat products		15 g	0.09
Cereals	White wheat and confectionery bread	35 g	0.06
	Cooked egg pasta	100 g	0.25
Fats	Butter, butter products, pork fat	5 g	0.03
	Margarine	5 g	0.31

Individuals were asked about the exact number of servings of products from groups specified in VIDEO-FFQ consumed per day/week/month. They were invited to indicate servings of consumed products and products added to consumed dishes (such data were important in the case of products which were elements of recipes for numerous dishes, e.g., eggs, fats). In the questionnaire, the participants indicated the typical number of servings of each product (not only integers, but also decimal parts).

During analysis, in order to obtain the daily number of servings, the total number of servings in the case of products specified per week or per month, was divided per seven or per 30 days. The Vitamin D intake from each product was estimated using the following equation: Vitamin D intake (μg) = daily number of servings × typical Vitamin D content in one serving. The total daily dietary Vitamin D intake was obtained as the sum of the Vitamin D intake values from all the analysed groups of products.

In the case of groups comprising fresh and smoked fish, as well as fish products, an additional question was included in the VIDEO-FFQ. Namely, the participants were asked to indicate the most commonly chosen products from the groups of fresh and smoked fish and from the groups of fish products. As a consequence, average Vitamin D content in the serving, individualised for each participant (obtained as a mean value for the indicated most commonly chosen products), was taken into account for the mentioned products.

### 2.2. Recruitment of Participants to the Validation of the VIDEO-FFQ

The VIDEO-FFQ was validated among a group of young women. The invitation to participate in the validation of VIDEO-FFQ, as well as all the required information regarding inclusion criteria was announced in social media (Internet social networking service). Participants received all the required information during recruitment and the further validation process, not only in oral format, but also in writing. The inclusion criteria were as follows: women aged 20–30 years, living in Warsaw, without on-going body mass reduction or not on any special diet, not pregnant or breastfeeding, with no chronic diseases. Eighty-seven individuals meeting the inclusion criteria volunteered to participate in the study and gave their informed consent to participate. The participants were informed about the possibility of withdrawal at any time with no consequences (also without specifying the reasons for their decision). Finally, the validation of assessed VIDEO-FFQ was conducted on a group of 75 young women, as 12 of the initially registered individuals did not complete all the required elements.

### 2.3. Validation of the VIDEO-FFQ

The validation study was conducted for three months—from September to November 2014. During this period, participants were asked to keep a three-day dietary record and to fill out the VIDEO-FFQ twice (FFQ1—filled out immediately after keeping the three-day dietary record and FFQ2—filled out six weeks after FFQ1). While completing VIDEO-FFQ, participants were asked about their average frequency of consumption throughout the last year. Both the three-day dietary record and VIDEO-FFQ assessment were based on the self-reported data. The participants were also asked about Vitamin D supplements—the supplement taken, the manufacturer, dose size and frequency.

Assessment of the obtained VIDEO-FFQ included an analysis of the validity (external validation compared with the results of the three-day dietary record, whereas both assessments were conducted by the same researcher) and reproducibility of the method (internal validation comparing results obtained twice—FFQ1 and FFQ2, with both assessments conducted by the same researcher), as defined by Willett and Lenart [26].

In the case of the three-day dietary record, the basis for the analysis constituted a record conducted during three typical, random non-consecutive days (two weekdays and one weekend day). The dietary record was conducted on the basis of widely accepted and applied rules—participants were asked to note all consumed food products and beverages, with the quantities specified using the kitchen scale (if participants possessed one) or using descriptive household serving sizes. Participants were instructed to record food products and dishes, in as much detail as possible, in the blank food diary provided. Individuals were asked to divide dishes into individual food products. Participants were asked to indicate weight of the products when commercial products were consumed. To provide reliable estimates of food intake, participants were instructed regarding the principles of making the dietary record, as well as about the required accurate and scrupulous recording of all consumed food products and beverages. The serving sizes were verified after completion of the records by a dietician using the Polish atlas of portion sizes of food products and dishes [25]. Vitamin D intake was analysed using the Polish dietician software “Dietetyk 2” (National Food and Nutrition Institute, Warsaw, Poland, 2001) and the Polish base of nutritional value of the products [24].

### 2.4. Statistical Analysis

Statistical analysis of validation included four elements:

(1) Calculating standard errors of estimation and median differences of Vitamin D intake in the assessment of validity (FFQ1 *vs.* three-day record and FFQ2 *vs.* three-day record) and in the assessment of reproducibility (FFQ1 *vs.* FFQ2)
(2) Assessment of the percentages of individuals classified into the same tertile of Vitamin D intake and individuals misclassified (classified into opposite tertiles of Vitamin D intake) in the assessment of validity (FFQ1 *vs.* three-day record and FFQ2 *vs.* three-day record) and in the assessment of reproducibility (FFQ1 *vs.* FFQ2)
(3) Analysis of the correlations between results obtained in the assessment of validity (FFQ1 *vs.* three-day record and FFQ2 *vs.* three-day record) and in the assessment of reproducibility (FFQ1 *vs.* FFQ2)—the normality of distribution of the results was analysed using the Shapiro-Wilk test, and, afterwards, Spearman’s rank correlation was applied for nonparametric distribution
(4) Analysis of the Bland-Altman plots in the assessment of validity (FFQ1 *vs.* three-day record and FFQ2 *vs.* three-day record) and in the assessment of reproducibility (FFQ1 *vs.* FFQ2)—the results were interpreted using the Bland-Altman index, whereas the limits of agreement value (LOA) were calculated as the sum of the mean absolute difference of Vitamin D intake measured by two methods, and the ±standard deviation of the absolute difference of Vitamin D intake recorded for two methods magnified by 1.96. In the analysis conducted with the Bland-Altman method to assess agreement between measurements, the Bland-Altman index of a maximum of 5% (95% of individuals observed to be beyond the LOA) was interpreted, as commonly assumed [27], as positive validation of the method of measurement

Moreover, the calculated Vitamin D intake obtained using the twice conducted VIDEO-FFQ (FFQ1, FFQ2) and three-day dietary record were compared for each participant with the recommended dietary intake (5.0 μg of cholecalciferol), according to the Polish recommendations on the AI level [28]. Simultaneously, the calculated Vitamin D intake was compared with the intake recommended by the Institute of Medicine at EAR (10.0 μg of cholecalciferol) and RDA levels (15.0 μg of cholecalciferol) [9].

In the comparison of the two methods (FFQ1 *vs.* three-day record; FFQ2 *vs.* three-day record; FFQ1 *vs.* FFQ2), the percentages of individuals from the same or from the conflicting category, according to Polish recommendations at the AI level, were assessed. The same category was interpreted as adequate or inadequate intake levels for both methods. The conflicting category was interpreted as contradictory intake levels (adequate and inadequate) for the compared methods.

A level of significance at *p* ≤ 0.05 was accepted. Statistical analysis was carried out using the Statistica software version 8.0 (StatSoft, Inc., Tulsa, OK, USA) and the Bland-Altman Statistica software macro by Matt Coates version 2009 (StatSoft, Inc., Tulsa, OK, USA).

## 3. Results

The Vitamin D intake observed for the analysed group calculated using a three-day dietary record and VIDEO-FFQ conducted twice (FFQ1, FFQ2) is presented in Table 3. The observed Vitamin D intake for the majority of the group was inadequate, *i.e.*, in each applied assessment over 85% of the group was characterised by intake values lower than the recommended 5.0 μg of cholecalciferol per day according to Polish recommendations at the AI level. Simultaneously, comparison with EAR recommendations of the Institute of Medicine revealed an intake lower than the recommended 10.0 μg of cholecalciferol per day was stated for over 98%, over 98%, and over 97% of the group in the case of FFQ1, FFQ2, and the three-day dietary record, respectively. The results obtained were also compared with RDA recommendations of the Institute of Medicine and an intake lower than the recommended 15.0 μg of cholecalciferol per day was observed for 100%, over 98%, and over 97% of the group in the case of FFQ1, FFQ2, and the three-day dietary record, respectively. Moreover, less than 7% of individuals declared taking Vitamin D supplements.

**Table 3:** The Vitamin D intake for the analysed group calculated using three-day dietary record and VIDEO-FFQ conducted twice (FFQ1, FFQ2), accompanied by percentages of individuals characterized by adequate or inadequate intake in comparison with the Polish recommendations at the AI level [28].

Category		3-Day Dietary Record	FFQ1	FFQ2
Mean (μg)		2.57	3.24	3.25
SD (μg)		3.04	2.13	3.11
Median (μg)		1.58 *	2.70 *	2.68 *
Minimum (μg)		0.02	0.53	0.36
Maximum (μg)		17.2	12.9	26.5
Percentage of individuals characterised by	adequate intake (%)	12.0	13.3	12.0
	inadequate intake (%)	88.0	86.7	88.0

* distribution different than normal (verified using Shapiro-Wilk test—*p* ≤ 0.05); FFQ1—VIDEO-FFQ filled out immediately after conducting a three-day dietary record; FFQ2—VIDEO-FFQ filled out 6 weeks after FFQ1.

The calculated standard errors of Vitamin D estimation in the assessment of validity for analysed VIDEO-FFQ in references to results of the three-day dietary record were 2.95 μg and 3.76 μg of cholecalciferol, for FFQ1 and for FFQ2, respectively. Simultaneously, the median differences of Vitamin D intake in reference to the three-day dietary record were 46.5% and 53.9% in the case of FFQ1 and FFQ2, respectively.

In the assessment of reproducibility, standard error of Vitamin D estimation was 2.93 μg of cholecalciferol. The median difference of Vitamin D intake was 13.6% for comparison between FFQ1 and FFQ2.

The percentages of individuals classified into the same tertile in the validation of the VIDEO-FFQ are presented in Table 4. The highest percentage of individuals classified into the same category (74.7%) accompanied by the lowest percentage of misclassified individuals (1.3%) was identified for comparison between FFQ1 and FFQ2 (assessment of reproducibility). FFQ1 was characterised by higher validity than FFQ2 in the tertile classification, but, simultaneously, FFQ2 was characterised by lower percentage of conflicting Vitamin D intake adequacy category than FFQ1.

**Figure 1:**
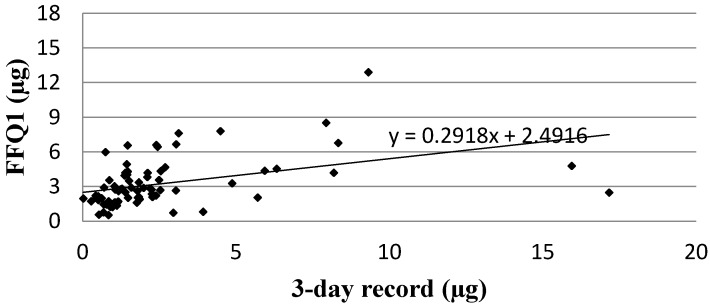
Analysis of correlation between VIDEO-FFQ1 and the three-day dietary record for Vitamin D daily intake (Spearman rank correlation coefficient; *p* < 0.01, R = 0.5402). FFQ1—VIDEO-FFQ filled out immediately after conducting the three-day dietary record.

Analysis of the correlation between FFQ1 and the three-day dietary record for Vitamin D daily intake is presented in Figure 1. The Spearman rank correlation coefficient (*p* < 0.01, R = 0.5402) revealed statistically significant association for Vitamin D daily intakes obtained using a validated method of VIDEO-FFQ (FFQ1) and the three-day dietary record.

**Figure 2:**
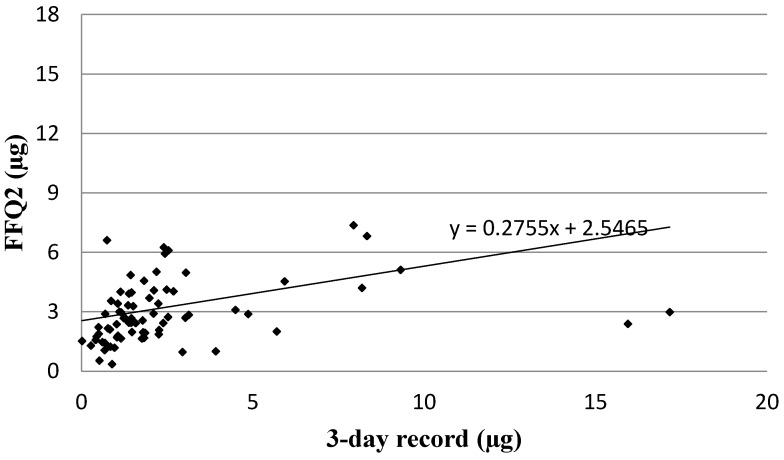
Analysis of correlation between VIDEO-FFQ2 and the three-day dietary record for Vitamin D daily intake (Spearman rank correlation coefficient; *p* < 0.01, R = 0.5025). FFQ2—VIDEO-FFQ filled out six weeks after FFQ1.

Analysis of the correlation between FFQ2 and the three-day dietary record for Vitamin D daily intake is presented in Figure 2. The same association was observed for FFQ2 as for FFQ1 when the Spearman rank correlation coefficient was applied (*p* < 0.01, R = 0.5025).

**Table 4:** The numbers and percentages of individuals classified into the same tertile and misclassified, accompanied by the numbers and percentages of individuals from the same or conflicting Vitamin D intake adequacy category presented for three-day dietary record and VIDEO-FFQ conducted twice (FFQ1, FFQ2) compared in pairs.

Category		Number	FFQ1 *vs.* 3-Day Dietary Record	FFQ2 *vs.* 3-Day Dietary Record	FFQ1 *vs.* FFQ2
		Percentage of Individuals			
Individuals classified into the same tertile		*n*	45	37	56
		%	60.0	49.3	74.7
Individuals misclassified (classified into opposite tertiles)		*n*	4	8	1
		%	5.3	10.7	1.3
Individuals of the	same Vitamin D intake adequacy category	*n*	62	64	68
		%	82.7	85.3	90.7
	conflicting Vitamin D intake adequacy category	*n*	13	11	7
		%	17.3	14.7	9.3

FFQ1—VIDEO-FFQ filled out right after conducting 3-day dietary record; FFQ2—VIDEO-FFQ filled out six weeks after FFQ1.

**Figure 3:**
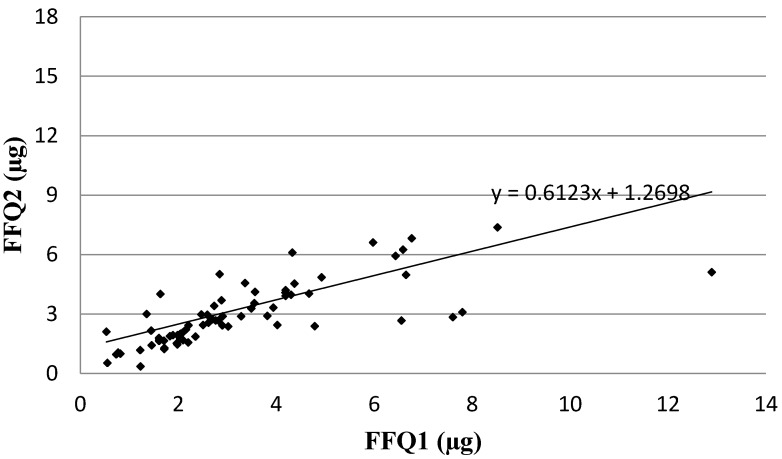
Analysis of correlation between VIDEO-FFQ1 and VIDEO-FFQ2 of Vitamin D daily intake (Spearman rank correlation coefficient; *p* < 0.01, R = 0.8235). FFQ1—VIDEO-FFQ filled out immediately after conducting the three-day dietary record; FFQ2—VIDEO-FFQ filled out six weeks after FFQ1.

Analysis of the correlation between FFQ1 and FFQ2 of Vitamin D daily intake is presented in Figure 3. In the assessment of reproducibility, similarly as in the assessment of validity, the Spearman rank correlation coefficient revealed a statistically significant association for Vitamin D daily intake obtained using VIDEO-FFQ between FFQ1 and FFQ2 (*p* < 0.01, R = 0.8235).

**Figure 4:**
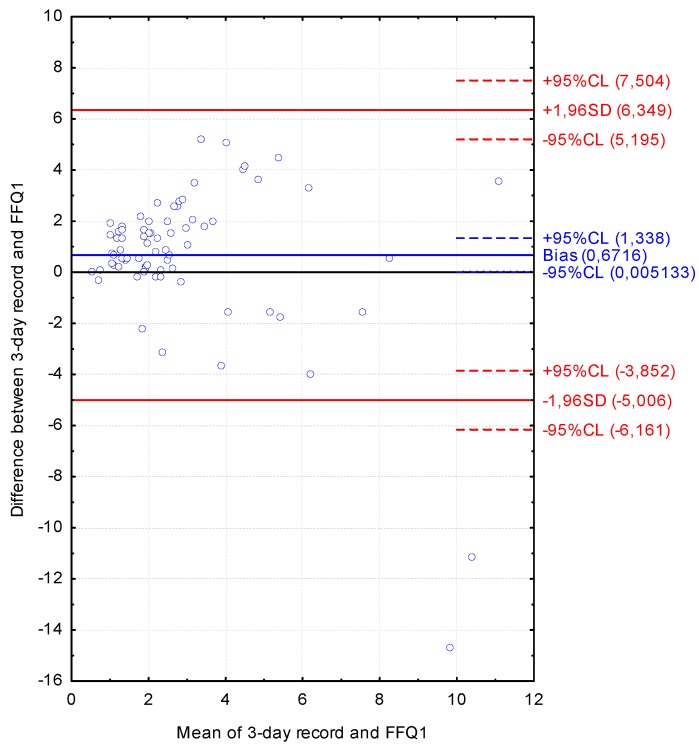
Bland-Altman plot comparing VIDEO-FFQ1 with the three-day dietary record for Vitamin D daily intake (Bland-Altman index of 2.7%). FFQ1—VIDEO-FFQ filled out immediately after conducting the three-day dietary record.

The Bland-Altman plot comparing FFQ1 with the three-day dietary record for Vitamin D daily intake is presented in Figure 4. The mean absolute difference of Vitamin D intake was observed to amount to 0.672. The interval from −5.006 (lower agreement limit) to 6.349 (upper agreement limit) was obtained for the LOA after adding a ±1.96-fold standard deviation. The number of individuals observed to be beyond the LOA value was 73 out of 75, corresponding to the Bland-Altman index of 2.7%.

**Figure 5:**
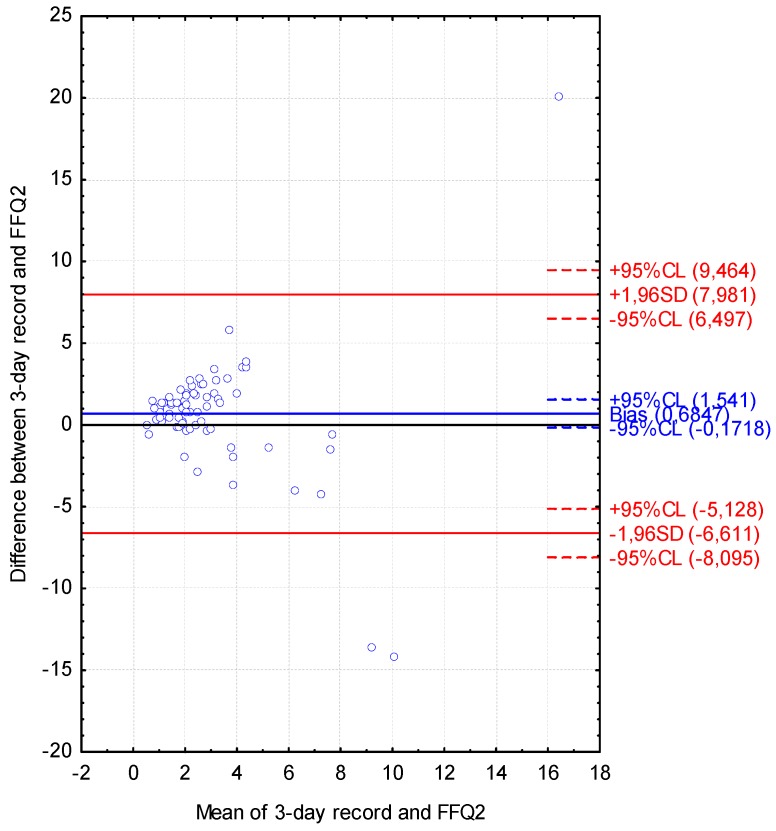
Bland-Altman plot comparing VIDEO-FFQ2 with the three-day dietary record for Vitamin D daily intake (Bland-Altman index of 4.0%); FFQ2—VIDEO-FFQ filled out six weeks after FFQ1.

The Bland-Altman plot comparing FFQ2 with the three-day dietary record for Vitamin D daily intake is presented in Figure 5. The mean absolute difference of Vitamin D intake was observed to amount to 0.685. The interval from −6.611 (lower agreement limit) to 7.981 (upper agreement limit) was obtained for the LOA after adding a ±1.96-fold standard deviation. The number of individuals observed to be beyond the LOA value was 72 out of 75, corresponding to the Bland-Altman index of 4.0%.

**Figure 6:**
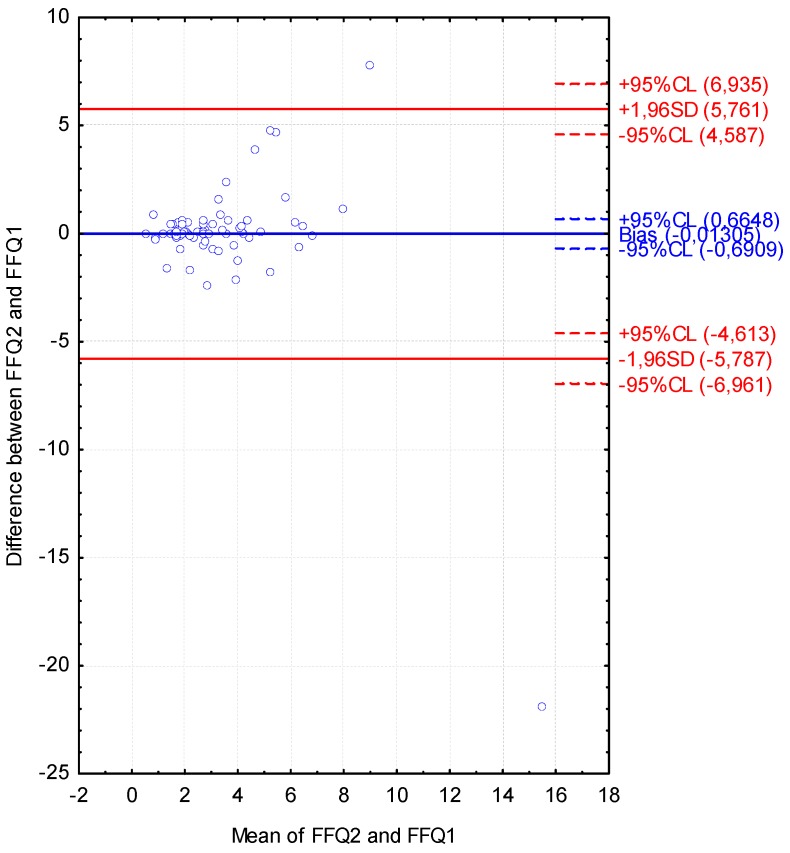
Bland-Altman plot comparing VIDEO-FFQ2 with VIDEO-FFQ1 of Vitamin D daily intake (Bland-Altman index of 2.7%). FFQ1—VIDEO-FFQ filled out immediately after conducting the three-day dietary record; FFQ2—VIDEO-FFQ filled out six weeks after FFQ1.

The Bland-Altman plot comparing FFQ2 with FFQ1 of Vitamin D daily intake is presented in Figure 6. The mean absolute difference of Vitamin D intake was observed to amount to −0.013. The interval from −5.787 (lower agreement limit) to 5.761 (upper agreement limit) was obtained for the LOA after adding a ±1.96-fold standard deviation. The number of individuals observed to be beyond the LOA value was 73 out of 75, corresponding to the Bland-Altman index of 2.7%.

The required value of less than 5.0% for the Bland-Altman index was achieved both for the assessment of validity (FFQ1 *vs.* three-day record and FFQ2 *vs.* three-day record) and in the assessment of reproducibility (FFQ1 *vs.* FFQ2).

## 4. Discussion

In Poland, the inadequate intake of Vitamin D among young women is commonly reported, and, according to various studies, 71% [29] to 89% [30] of young women were characterised by an intake below the recommended value. The corresponding results were observed in the case of the presented study, as the observed Vitamin D intake for the majority of the group was inadequate. For the cut-off of 5.0 μg of cholecalciferol per day, over 85% of the group was characterised by intake values lower than those of Polish recommendations [28]. However, as the report of Institute of Medicine [9] indicated, the cut-off value of 10.0 μg of cholecalciferol per day for EAR level, the problem of inadequate intake of Vitamin D in a group of young Polish women may be even more serious, as for over 97% of the surveyed group, intake lower than recommended 10.0 μg was stated.

Vitamin D is a nutrient that is obtained from a limited number of products in the Polish diet, as the richest sources of Vitamin D are fish and fish products, including fish oil, and are the only products available in Poland characterised by a significant percentage of this nutrient [31]. In spite of such a serious problem concerning the inadequate intake of Vitamin D in young women and a limited number of Vitamin D sources, no dedicated Vitamin D food frequency questionnaire has been designed and applied in Poland so far. However, it must be mentioned that Kowalkowska *et al.* [32] assessed the Full Food-Frequency Questionnaire with a list of 165 products and dishes that allowed an estimation of the intake of Vitamin D and 30 other nutrients, and an analysis of the Bland-Altman index for Vitamin D revealed values not higher than 5%. Similarly, a semi-quantitative food frequency questionnaire including 95 food groups and products, applied by Sochacka-Tatara and Pac [33] to calculate intake of 23 nutrients, allowed an assessment of the Vitamin D intake, whereas the LOA was stated to be good. Woźniewicz *et al.* [34] also assessed the intake of products that are a source of Vitamin D using a food frequency questionnaire, but only the number of servings of products containing Vitamin D, not its intake, was calculated.

The recent systematic review of the validation methods for measuring continuous variables indicated the Bland-Altman method as the most common, and it was shown that a correlation coefficient and means comparison were also frequently applied [35]. In the case of the validation studies of Vitamin D food frequency questionnaires, the comparison of the percentages of individuals classified into the same tertile [20] or quartile [21] was frequently used as well, so this method of comparison of the results was also used in the validation. However, the Bland-Altman plot, being the recommended approach to compare results obtained using various methods, is still indicated as the “gold standard” of validation, and it should be the basis for the validation of food frequency questionnaires [30]. As a consequence, the results of the analysis of the Bland-Altman plot should be treated as the predominant ones of the obtained validation results.

In the research of Park *et al.* [23], two questionnaires based on the Canadian Calcium Assessment Tool (CAT) [36] and the newly-developed Korean Calcium Assessment Tool (KCAT) were compared to assess calcium and Vitamin D intake. The CAT questionnaire was previously used during the Korean National Health and Nutrition Survey (KNHANES) [37]. However, it was concluded that in the case of the diet of Korean women, calcium intake is derived from different food products than for women from Western countries, since the main sources of calcium in Korea constitute not dairy but plant products [38]. As a consequence, the newly developed KCAT questionnaire included a number of specific plant products, such as fermented soy and soymilk, seaweed, kimchi, ramen or coffee mixes, which in Korea are a source of calcium and Vitamin D [23]. In the study by Park *et al.* [23], Vitamin D intake in the Korean population, assessed using the KCAT questionnaire, was significantly higher than that assessed using the CAT questionnaire, which may result from different food products being included.

In food frequency questionnaires designed for Western countries, differentiation is also required. In the case of questionnaires applied in the United States of America, it was also necessary to include low-fat and non-fat food products. Such a differentiation providing for fat content was applied in the Willett questionnaire developed to asses multiple nutrients adapted by Caan *et al.* [39] and afterwards applied for Vitamin D by Osowski *et al.* [16] as the Willett 97GP 2003 questionnaire. The food frequency questionnaire, being the modified version of previously reported questionnaires by Musgrave *et al.* [40] and Salamone *et al.* [41], applied in the research of Taylor *et al.* [17], focused on the assessment of calcium and Vitamin D intake and was designed in a similar way. The questionnaire included not only low-fat and non-fat products, but also fortified or frozen products, fast food meals, meal replacement formulas and sports nutrition bars [17].

As previously indicated, the majority of validated Vitamin D questionnaires was designed for populations in non-European countries with distinct consumption patterns present. However, inadequate Vitamin D intake frequency in Europe is very common, which is a serious public health issue [7,8]. The designed VIDEO-FFQ was developed based on typical European food products or groups of products. The aim of such design was to ensure the possibility of applying the questionnaire in various European countries and regions. Moreover, the modifications to the questionnaire are quite simple for food products that are fortified in some countries or regions, so the questionnaire may not only be applicable to European countries.

As Vitamin D is indicated as a nutrient with one of the highest prevalences of inadequate intake [8], some food frequency questionnaires from the United States of America are designed to calculate Vitamin D intake exclusively [18,19]. Such a possibility is also associated with the relatively limited range of Vitamin D food sources, resulting in the conciseness of the Vitamin D intake questionnaire in comparison with multiple nutrients questionnaires. In the study by Nucci *et al.* [19], two questionnaires were applied, namely the long food frequency questionnaire (LFFQ), designed by Harvard Medical School, and the short food frequency questionnaire (SFFQ), adapted from a questionnaire developed at Boston University’s Medical Center. The LFFQ was designed to assess the intake of 17 nutrients, including total energy, so the number of questions was substantial—participants were asked 152 questions regarding their intake of food products. The SFFQ was designed exclusively for Vitamin D, so only 17 questions about food products were included. The assessment of validity revealed a modest correlation between LFFQ and SFFQ for children (*p* < 0.001, R = 0.35 for baseline measurement; *p* < 0.001, R = 0.37 for follow-up measurement conducted after six months), but this correlation was significantly stronger in the case of Caucasian individuals (*p* < 0.001, R = 0.48 for baseline; *p* < 0.001, R = 0.49 for follow-up) [19]. It seems obvious that a short questionnaire increases the possibility of scrupulous completion, so even though Vitamin D intake values for SFFQ and LFFQ differed significantly, the obtained SFFQ was concluded by Nucci *et al.* [19] to be a reasonably valid and reproducible tool.

In the study by Hacker-Thompson *et al.* [18], the Vitamin D food frequency questionnaire—Brief Vitamin D Questionnaire (BVDQ), developed in the Clinical Research Center at the University of California—was compared with the Block Health History and Habits Questionnaire 1998 (BHHHQ98) and the three-day dietary record. Significant correlations for Vitamin D intake were observed between BVDQ and BHHHQ98 (*p* < 0.001, R = 0.51), as well as BVDQ and the three-day dietary record (*p* < 0.001, R = 0.43) in a group of postmenopausal women. Moreover, although the BHHHQ98 included 110 questions, regarding food products’ intake, whereas BVDQ only 26, the Bland-Altman analyses revealed that the results of both questionnaires were similar and that the mean difference in Vitamin D intake between them was 0.12 μg.

The studies of Vitamin D intake questionnaires revealed various levels of validity, but positive validation of methods of measurement [27] was confirmed for some of the published validations conducted using the Bland-Altman method, when a level of less than 5% for the Bland-Altman index was achieved or almost achieved. In the research of Park *et al.* [23], the comparison between the CAT and KCAT questionnaires revealed a Bland-Altman index of 3.1% calculated for women younger than 50 years and 3.9% for the general group of women. In the research of Pritchard *et al.* [21], the comparison between the food frequency questionnaire and a five-day dietary record indicated that the number of individuals observed to be beyond the LOA value (14 out of 15) corresponded to a Bland-Altman index of 6.7%. Simultaneously, in the research of Taylor *et al.* [17], analysis of the comparison between food frequency questionnaires and four-day dietary records obtained from healthy female adolescents revealed the number of individuals observed beyond the LOA value to be 71 out of 75, which corresponded to a Bland-Altman index of 6.3%.

In the presented validation of VIDEO-FFQ, Bland-Altman indexes were lower than in the previously mentioned studies comparing food frequency questionnaires with the dietary record. Such a difference may result from the fact that VIDEO-FFQ was designed only for Vitamin D, whereas questionnaires applied by Pritchard *et al.* [21] or Taylor *et al.* [17] were designed to assess the intake of calcium, Vitamin D and Vitamin K [21], or calcium and Vitamin D [17], respectively. As a consequence, the food frequency questionnaires applied in the study by Pritchard *et al.* [21] and Taylor *et al.* [17] contained 161 questions and 40 questions regarding intake of food products, respectively, whereas only 21 questions were included in the presented VIDEO-FFQ. It is well known that the number of questions is a very important factor in questionnaire design, as questionnaires that are too long can induce fatigue among respondents and provoke uniform or inaccurate answers, causing lower accuracy of results [42].

In the research of Wu *et al.* [20], similar to the presented study, the comparison of the percentage of individuals classified into the same tertile and individuals misclassified was conducted; however, two food frequency questionnaires were applied—the original, previously used by Shrestha *et al.* [43], and a modified one. The percentages of misclassified individuals for the original and modified questionnaires in comparison with the seven-day dietary record in the research of Wu *et al.* [20] were 12% and 17%, respectively. It was concluded that a food frequency questionnaire of this type aimed at assessing the Vitamin D intake is a valid tool and may provide a reasonable estimation of Vitamin D intake [20]. In the presented validation of VIDEO-FFQ, the percentages of misclassified individuals for FFQ1 and FFQ2 in comparison with the dietary record were even lower, amounting to 5.3% and 10.7%, respectively.

It is observed, in the validation of food frequency questionnaires, that coefficients higher than 0.7 are rare in the analysis of correlation, while such phenomena are called the “ceiling of validity” and are attributed to the fact that the inherent complexity of the human diet cannot be fully captured by a structured questionnaire [17]. Based on this fact, the obtained correlation coefficients R = 0.5402 (for correlation between FFQ1 and three-day dietary records) and R = 0.5025 (for correlation between FFQ2 and three-day dietary records)—should be interpreted as satisfactory. Moreover, even a higher correlation coefficient of the amount of 0.8235 was obtained in the case of a correlation between FFQ1 and FFQ2.

The number of individuals participating in the presented validation of VIDEO-FFQ may be indicated as a certain limitation of the presented study. In spite of the fact that VIDEO-FFQ was positively validated in a group of young women, further validation in larger groups should be conducted.

It must be concluded that, based on the presented validation, the VIDEO-FFQ (presented in Table 1) is a tool positively assessed on a group of young women, characterised by a satisfactory level of validity and reproducibility. After essential modifications, VIDEO-FFQ may also be used in European countries other than Poland. Taking into account the fact that the natural Vitamin D content in animal products, in spite of some minor differences between countries, is generally similar [44], the main differences that must be taken into account are associated with the fortification of food products applied in some countries or regions. The VIDEO-FFQ may be recommended as a practical quick method of assessment of Vitamin D intake adequacy in the European population of young women and is a promising method for other populations.

## 5. Conclusions

1. Vitamin D intake for the majority of surveyed Polish young women is inadequate, as over 85% of them are characterised by intake values lower than 5.0 μg per day and over 97% by intake values lower than 10.0 μg of cholecalciferol per day.
2. It is possible to design a reliable food frequency questionnaire aimed at assessing Vitamin D intake, characterised by high accuracy due to the limited number of questions.
3. The Bland-Altman indexes for validated VIDEO-FFQ were lower than 5%, which may be interpreted as a positive assessment of its validity and reproducibility.
4. The VIDEO-FFQ may be indicated as a practical tool for the estimation of Vitamin D intake in the population of young women.

## References

[1] Calvo M.S., Whiting S.J., Barton C.N. (2005). Vitamin D intake: A global perspective of current status. J. Nutr..

[2] European Food Safety Authority (EFSA) (2012). Scientific opinion on the tolerable upper intake level of vitamin D. EFSA J..

[3] Paturi M., Tapanainen H., Reinivuo H., Pietinen P. (2008). The National FINDiet 2007 Survey.

[4] Becker W., Pearson M. (2002). Riksmaten 1997–1998. Befolkningens Kostvanor och Näringsintag. Metod- Och Resultatanalys (Riksmaten 1997–1998. Dietary Habits and Nutrient Intake in Sweden. Benchmarking Analysis).

[5] Spiro A., Buttriss J.L. (2014). Vitamin D: An overview of vitamin D status and intake in Europe. Nutr. Bull..

[6] Flynn A., Hirvonen T., Mensink G.B., Ocke M.C., Serra-Majem L., Stos K., Szponar L., Tetens I., Turrini A., Fletcher R. (2009). Intake of selected nutrients from foods; from fortification and from supplements in various European countries. Food Nutr. Res..

[7] Elmadfa I., Meyer A., Nowak V., Hasenegger V., Putz P., Verstraeten R., Remaut-DeWinter A.M., Kolsteren P., Dostálová J., Dlouhý P. (2009). European Nutrition and Health Report 2009. Forum Nutr..

[8] Roman Viñas B., Ribas Barba L., Ngo J., Gurinovic M., Novakovic R., Cavelaars A., de Groot L.C., van’t Veer P., Matthys C., Serra Majem L. (2011). Projected prevalence of inadequate nutrient intakes in Europe. Ann. Nutr. Metab..

[9] Institute of Medicine, Committee to Review Dietary Reference Intakes for Vitamin D and Calcium, Food and Nutrition Board (2011). Dietary Reference Intakes for Calcium and Vitamin D.

[10] Ross A.C., Manson J.E., Abrams S.A., Aloia J.F., Brannon P.M., Clinton S.K., Durazo-Arvizu R.A., Gallagher J.C., Gallo R.L., Jones G. (2011). The 2011 report on dietary reference intakes for calcium and vitamin D from the Institute of Medicine: What clinicians need to know. J. Clin. Endocrinol. Metab..

[11] Kennel K.A., Drake M.T., Hurley D.L. (2010). Vitamin D deficiency in adults: When to test and how to treat. Mayo Clin. Proc..

[12] LeFevre M.L., U.S. Preventive Services Task Force (2015). Screening for vitamin D deficiency in adults: U.S. Preventive Services Task Force recommendation statement. Ann. Intern. Med..

[13] Bailey R.L., Dodd K.W., Goldman J.A., Gahche J.J., Dwyer J.T., Moshfegh A.J., Sempos C.T., Picciano M.F. (2010). Estimation of total usual calcium and vitamin D intakes in the United States. J. Nutr..

[14] Benjamin R.M. (2010). Bone health: Preventing osteoporosis. J. Am. Diet. Assoc..

[15] Sadat-Ali M., al Elq A.H., al-Turki H.A., al-Mulhim F.A., al-Ali A.K. (2011). Influence of vitamin D levels on bone mineral density and osteoporosis. Ann. Saudi Med..

[16] Osowski J.M., Beare T., Specker B. (2007). Validation of a food frequency questionnaire for assessment of calcium and bone-related nutrient intake in rural populations. J. Am. Diet. Assoc..

[17] Taylor C., Lamparello B., Kruczek K., Anderson E.J., Hubbard J., Misra M. (2009). Validation of a food frequency questionnaire for determining calcium and vitamin D intake by adolescent girls with anorexia nervosa. J. Am. Diet. Assoc..

[18] Hacker-Thompson A., Schloetter M., Sellmeyer D.E. (2012). Validation of a dietary vitamin D questionnaire using multiple diet records and the block 98 health habits and history questionnaire in healthy postmenopausal women in northern California. J. Acad. Nutr. Diet..

[19] Nucci A.M., Russell C.S., Luo R., Ganji V., Olabopo F., Hopkins B., Holick M.F., Rajakumar K. (2013). The effectiveness of a short food frequency questionnaire in determining vitamin D intake in children. Dermatoendocrinol.

[20] Wu H., Gozdzik A., Barta J.L., Wagner D., Cole D.E., Vieth R., Parra E.J., Whiting S.J. (2009). The development and evaluation of a food frequency questionnaire used in assessing vitamin D intake in a sample of health young Canadian adults of diverse ancestry. Nutr. Res..

[21] Pritchard J.M., Seechurn T., Atkinson S.A. (2010). A food frequency questionnaire for the assessment of calcium, vitamin D and vitamin K: A pilot validation study. Nutrients.

[22] Papandreou D., Rachaniotis N., al Mussabi W. (2014). Validation of a Food Frequency Questionnaire for vitamin D and calcium intake in healthy female college students. Food Nutr. Sci..

[23] Park Y., Kim S., Lim Y., Ha Y., Chang J., Kim J., Min Y., Chung H. (2013). Validation of new food frequency questionnaire for assesment of calcium and vitamin D intake in Korean women. J. Bone Metab..

[24] Kunachowicz H., Nadolna J., Przygoda B., Iwanow K. (2005). Food Composition Tables.

[25] Szponar L., Wolnicka K., Rychlik E. (2000). Atlas of Portion Sizes of Food Products and Dishes.

[26] Willett W., Lenart E., Willett W. (2013). Reproducibility and validity of food frequency questionnaires. Nutritional Epidemiology.

[27] Myles P.S., Cui J. (2007). Using the Bland-Altman method to measure agreement with repeated measures. Br. J. Anaesth..

[28] Jarosz M. (2012). Human Nutrition Recommendations for Polish Population.

[29] Przysławski J., Bolesławska O., Kaźmierczak A. (2012). An evaluation of the level of intake of selected vitamins among students in Poznan on the background of other studies. Bromatol. Chem. Toksykol..

[30] Wyka J., Żechałko-Czajkowska A. (2008). Vitamins and minerals in diets of first year female students of the Wrocław university of environmental and life sciences. Pol. J. Food Nutr. Sci..

[31] Głąbska D., Włodarek D., Włodarek D., Lange E., Kozłowska L., Głąbska D. (2014). Vitamins. Diet Therapy.

[32] Kowalkowska J., Slowinska M.A., Slowinski D., Dlugosz A., Niedzwiedzka E., Wadolowska L. (2013). Comparison of a full food-frequency questionnaire with the three-day unweighted food records in young Polish adult women: Implications for dietary assessment. Nutrients.

[33] Sochacka-Tatara E., Pac A. (2014). Relative validity of a semi-quantitative FFQ in 3-year-old Polish children. Public Health Nutr..

[34] Woźniewicz M., Jeszka J., Sadowska K., Bajerska J. (2009). Frequency of consumption of products and foods—Sources of vitamin D and calcium—Among secondary school students. EJPAU.

[35] Zaki R., Bulgiba A., Ismail R., Ismail N.A. (2012). Statistical methods used to test for agreement of medical instruments measuring continuous variables in method comparison studies: A systematic review. PLoS ONE.

[36] Hung A., Hamidi M., Riazantseva E., Thompson L., Tile L., Tomlinson G., Stewart B., Cheung A.M. (2011). Validation of a calcium assessment tool in postmenopausal Canadian women. Maturitas.

[37] Korea Centers for Disease Control and Prevention, Ministry of Health & Welfare (2008). Korea Health Statistics 2007: Korea National Health and Nutrition Examination Survey (KNHANESIV-1) Seoul.

[38] Park H.M., Heo J., Park Y. (2011). Calcium from plant sources is beneficial to lowering the risk of osteoporosis in postmenopausal Korean women. Nutr. Res..

[39] Caan B.J., Slattery M.L., Potter J., Quesenberry C.P., Coates A.O., Schaffer D.M. (1998). Comparison of the Block and the Willett self-administered semiquantitative food frequency questionnaires with an interviewer-administered dietary history. Am. J. Epidemiol..

[40] Musgrave K.O., Giambalvo L., Leclerc H.L., Cook R.A., Rosen C.J. (1989). Validation of a quantitative food frequency questionnaire for rapid assessment of dietary calcium intake. J. Am. Diet. Assoc..

[41] Salamone L.M., Dallal G.E., Zantos D., Makrauer F., Dawson-Hughes B. (1994). Contributions of vitamin D intake and seasonal sunlight exposure to plasma 25-hydroxyvitamin D concentration in elderly women. Am. J. Clin. Nutr..

[42] Choi B.C., Pak A.W. (2005). A catalog of biases in questionnaires. Prev. Chronic Dis..

[43] Shrestha R.K., Whiting S.J., Briggs L., Burak C. (1994). Use of a semiquantitative food frequency questionnaire to assess intake of calcium and vitamin D in children, adolescents, and young adults. Am. J. Clin. Nutr..

[44] Schmid A., Walther B. (2013). Natural vitamin D content in animal products. Adv. Nutr..

